# Supplementary material for: RAC1, a Potential Diagnostic and Prognostic Marker for Diffuse Large B Cell Lymphoma
Source: Cells. 2022 Dec 14;11(24):4039. doi: 10.3390/cells11244039 (PMC9776810; doi:10.3390/cells11244039)
Supplement: Supplementary file 1 [file cells-11-04039-s001.zip › cells-1965904-supplementary.pdf]

Table S1. Differential expression analyses of 17 Rho/Rac family genes between DLBCL tissues and normal tissues in TCGA-DLBC and GTEx datasets.

Rho/Rac family gene	Mean DLBCL	Mean Normal	Difference	P value
RHOA	8.362	7.821	0.541	0.001
RHOB	3.634	4.634	-1.000	2.000
RHOC	6.201	5.286	0.915	0.000
RHOD	1.304	0.304	1.000	0.000
RHOF	5.525	3.789	1.736	0.000
RHOG	7.359	8.086	-0.727	0.000
RHOJ	1.272	0.098	1.174	0.000
RHOQ	4.743	3.983	0.760	0.001
RHOU	1.480	1.114	0.366	0.000
RHOV	1.133	1.096	0.037	0.000
RAC1	7.619	6.420	1.199	0.000
RAC2	8.646	8.765	-0.119	0.184
RAC3	1.853	0.801	1.052	0.000
CDC42	6.987	6.606	0.381	0.107
RND1	1.683	0.642	1.041	0.000
RND2	0.179	0.048	0.131	0.000
RND3	2.315	0.289	2.026	0.000

Wilcoxon rank sum test was used to evaluate the significant difference of Rho/Rac family gene expression levels in different groups.

Table S2. Receiver operating characteristic curve analyses of Rho/Rac subfamily members between normal tissues and DLBCL tissues in TCGA dataset.

Rho/Rac subfamilies	Gene	AUC	Cut-off Value	Sensitivity	Specificity
RHO subfamily	RHOA	0.652	7.230	0.979	0.279
	RHOB	0.640	4.943	0.936	0.381
	RHOC	0.666	5.283	0.936	0.543
RND subfamily	RND1	0.840	0.637	1.000	0.667
	RND2	0.869	0.063	0.809	0.766
	RND3	0.968	0.807	0.957	0.928
	RHOD	0.936	0.580	0.915	0.867
	RHOF	0.837	4.022	0.936	0.595
RAC subfamily	RAC1	0.877	6.961	0.957	0.705
	RAC2	0.559	9.205	0.809	0.356
	RAC3	0.869	1.202	0.872	0.725
	RHOG	0.692	7.816	0.894	0.523
CDC42 subfamily	CDC42	0.571	6.136	0.894	0.322
	RHOJ	0.989	0.400	0.979	0.953
	RHOQ	0.650	3.652	0.915	0.426
	RHOU	0.665	0.766	0.894	0.435
	RHOV	0.736	0.144	0.936	0.700

Table S3. Co-expression correlation analyses of BTK and Rho/Rac family genes in DLBCL tissues based on TCGA-DLBC dataset.

Target gene	Rho/Rac family gene	Correlation coefficient	P value (Spearman)
BTK	RHOA	0.151	0.304
BTK	RHOB	0.191	0.192
BTK	RHOC	-0.239	0.101
BTK	RND1	-0.157	0.286
BTK	RND2	-0.2	0.173
BTK	RND3	-0.038	0.796
BTK	RHOD	0.069	0.641
BTK	RHOF	0.349	0.015
BTK	RAC1	-0.355	0.014

BTK	RAC2	0.449	0.002
BTK	RAC3	-0.001	0.993
BTK	RHOG	-0.247	0.091
BTK	RHOJ	0.312	0.032
BTK	RHOQ	0.342	0.018
BTK	RHOU	0.105	0.476
BTK	RHOV	-0.173	0.238
BTK	CDC42	0.482	<0.001

Table S4. Differential expression analyses of RAC1 in multiple tumor tissues and adjacent normal tissues in TCGA pan-cancer datasets.

Cancer Type	Median DLBC	Median Normal	Difference	P value
BLCA	8.786	8.252	0.534	0.003
BRCA	8.534	8.309	0.225	0.000
CHOL	8.171	6.196	1.975	0.004
COAD	8.430	8.313	0.117	0.090
ESCA	9.007	7.893	1.114	0.023
HNSC	8.593	8.200	0.393	0.000
KICH	7.470	8.111	-0.641	0.000
KIRC	8.296	7.837	0.459	0.000
KIRP	8.442	7.587	0.855	0.000
LIHC	7.290	6.398	0.892	0.000
LUAD	8.545	8.220	0.325	0.000
LUSC	8.559	8.171	0.388	0.000
PAAD	8.913	8.472	0.441	0.125
PRAD	8.076	8.162	-0.086	0.699
READ	8.473	8.332	0.141	0.203
STAD	8.520	7.986	0.534	0.000
THCA	8.109	7.974	0.135	0.014
UCEC	8.145	8.276	-0.131	0.025

Wilcoxon rank sum test was used to evaluate the significant difference of RAC1 expression levels in different groups.

Table S5. Differential expression analyses of RAC1 in different clinical stages of patients with DLBCL in TCGA dataset.

Cancer Type	Median Stage I and II	Median Stage III and IV	Difference	P value
DLBC	7.955	7.603	0.352	0.012

Wilcoxon rank sum test was used to evaluate the significant difference of RAC1 expression levels between two groups.
